# Influence of egg card color preference, inoculation time, generational succession, and learning experience on the parasitism of *Trichogramma ostriniae*

**DOI:** 10.1371/journal.pone.0315970

**Published:** 2025-02-21

**Authors:** Huixian Wang, Wenya Zhu, Ruifeng Guo, Ye Zhang

**Affiliations:** 1 Shanxi Agricultural University, Taiyuan, China; 2 College of Plant Protection, Shanxi Agricultural University, Taiyuan, China; 3 Institute of Sorghum, Shanxi Agricultural University, Jinzhong, China; Forest Survey of India, INDIA

## Abstract

This study investigated the parasitic selection preferences of female *Trichogramma ostriniae* on egg cards of different colors, considering the effects of inoculation time, generations, and learning experiences on wasp parasitism. The preferences of female *T*. *ostriniae* for oviposition were assessed across egg cards of different colors. Subsequent experiments identified the egg card background color most preferred by *T*. *ostriniae* and examined the influence of inoculation time, generations, and learning experiences on parasitism rates. The findings revealed a significant preference for *T*. *ostriniae* for yellow egg cards compared to other colors, with the highest parasitism rate observed on yellow and the lowest on red. Inoculation time had a significant effect on the parasitism of *T*. *ostriniae* on yellow egg cards, with the highest parasitism observed at 16h post-inoculation and a noticeable negative linear correlation with increasing inoculation time up to 48h (F = 8.73, P<0.05). The number of generations did not significantly affect the parasitism rate of *T*. *ostriniae* on yellow egg cards, nor did the learning experiences show a significant difference in the relative parasitism of *T*. *ostriniae* on yellow egg cards among the learning experiences. However, T. ostriniae had a significantly higher parasitization rate on yellow egg cards compared to white egg cards, regardless of generation or learning experience. Those results showed that female *T*. *ostriniae* strongly prefers yellow. The highest parasitism of female *T*. *ostriniae* on yellow egg cards was observed at 16h post-inoculation.

## Introduction

*Trichogramma*, a minute egg-parasitizing insect from the family Trichogrammatidae within the order Hymenoptera, holds significant importance in biological pest control [1,2]. This genus has achieved notable success as an effective biological control agent in modern agricultural and forestry pest management strategies [3,4]. Its success is largely due to the ease of rearing on alternative hosts in the laboratory and its broad spectrum of pest targets, making *Trichogramma spp*. pivotal in integrated pest management [5]. Among these, *Trichogramma ostriniae* (Pang & Chen) is widely used as a dominant parasitoid of Asian corn borer (*Ostrinia furnacalis*) eggs [2]. In China, *T*. *ostriniae* is the primary parasitoid of corn borer eggs [6]. Mass production and timely release of this species have substantially reduced the use of chemical insecticides, which play crucial roles in maintaining agroecological environments.

Coloration has played a pivotal role in deterring phytophagous insects and attracting natural enemies throughout the evolutionary history of complex natural ecosystems and tritrophic interactions [7]. Pak and de Jong observed that *T*. *buesi* exhibits an adaptive preference for egg colors matching specific hosts, such as *Mamestra brassicae* and *Pieris brassicae*, in Dutch cabbage fields [8]. *T*. *buesi* preferred white over yellow painted beads, aligning with the suitability of white *M*. *brassicae* eggs for wasp development, compared to the unsuitable yellow *P*. *brassicae* eggs. In a non-target risk assessment for *T*. *ostriniae* in northeastern U.S. cornfields, Wright *et al*. (2005) identified the rustic shoulder-knot moth (Apamea sordens) as having a higher risk of impact due to its frequent presence in corn habitats [9]. However, since *A*. *sordens* lays green eggs (M.P. Hoffmann, unpublished), the risk could be mitigated by *T*. *ostriniae*’s low preference for green eggs. Lobdell *et al*. (2005) investigated the color preferences of *T*. *ostriniae*, the Asian corn borer egg parasitoid, in proximate host-searching behaviors. They discovered a pronounced female preference towards yellow and white model eggs, with variations in search and parasitism activities observed across different colors [10]. In contrast, Lukianchuk and Smith observed no significant difference in the parasitism behavior of *Trichogramma minutum* when presented with eggs against white and green backgrounds, suggesting that color did not affect their parasitic behavior [11]. Research on the color preferences of trichogrammatids is currently limited, with most previous studies focusing on the impact of host egg coloration, habitat hues, and food color on their search and parasitism behaviors. These studies often overlook the impact of egg cards of different colors on insect preferences. Therefore, our study aimed to assess the preferences of female *T*. *ostriniae* for egg cards in seven colors: white, yellow, orange, pink, red, blue, and green. Additionally, we investigated the parasitism behavior of the *T*. *ostriniae* on egg cards of the preferred color. By identifying the colors that facilitate host localization by the *T*. *ostriniae*, we sought to reveal how these preferred colors influence their parasitism efficiency. Ultimately, this study provides a theoretical basis for enhancing the potential of the *T*. *ostriniae* in pest control applications in the field.

## Materials and methods

### Parasitoid wasps and host insects

The parasitoid wasp *T*. *ostriniae* was collected from the parasitized eggs of the corn borer in Xinzhou City, Shanxi Province (longitude 112°52’15.64’’E, latitude 38°27’16.60’’N). Permission to access fields was obtained from all farmers. After field collection, the species was purified through single-wasp isolation and male genitalia dissection in the laboratory, with species identification confirmed by Professor Yuanxi Li from Nanjing Agricultural University. To establish stable indoor breeding colonies, *Corcyra cephalonica (Stainton)* eggs were used as hosts for several generations. The rearing conditions included a temperature of 25°C, 75% relative humidity, and a 15 L: 9 D photoperiod. *T*. *ostriniae* were fed a solution of 20% honey water, while *C*. *cephalonica* was fed a mixture of wheat bran mixed with soybean, corn, and wheat flour. Fresh and clean *C*. *cephalonica* eggs obtained within 24h were irradiated with a 30w ultraviolet lamp for 30 min to kill the embryos before being used to breed *T*. *ostriniae*.

### Preparation of parasitic egg cards

The A4 colored card (M&G, Shanghai, China) was purchased from Taiyuan Deyi Culture Square. It measures 21 cm × 29.7 cm and includes white, yellow, orange, pink, red, blue, and green colors. The color parameters for each card are detailed in S1 Table.

### Selection of different color cardboard by *T*. *ostriniae*

To create the egg cards, inactivated fresh *C*. *cephalonica* eggs were evenly scattered on white latex-coated cardboard to achieve a density of 500 eggs per colored zone. Egg cards with different colored backgrounds were prepared by cutting colored cardstock into rectangles measuring 1.3 cm × 3 cm. Following this, the colored rectangles were paired with white cardstock to create dichromatic combinations: white yellow, white orange, white pink, white red, white blue, and white green. Each color pair was separated by an intercard spacing of 0.4 cm. A finger tube was inverted over individual *T*.*ostriniae* wasps to utilize their positive geotaxis, facilitating their movement onto the inner wall of the tube. Under a stereomicroscope, female wasps were carefully selected and transferred using a soft brush into a container. Female *T*. *ostriniae* (five wasps/box) that had emerged within the last 24h were selected and inserted into a rectangular box (L×H×W = 15 cm×4.5 cm×10 cm) with an opening at the top, surrounded by opaque material to protect it from light, with the bottom also shielded from light. Next, the top opening of the box was sealed with white-yellow, white-orange, white-pink, white-red, white-blue, and white-green egg cards, respectively, and parasitized at a temperature of 25°C, relative humidity of 75%, and a photoperiod of 14 L:10D for 24h. Subsequently, the female wasps were removed and the parasitized eggs were further incubated under the same conditions for an additional 24 h. Following the completion of parasitism, all parasitizeda eggs were allowed to darken and examined microscopically. The number of blackened eggs on the egg cards with different colored backgrounds was counted. Each experiment was repeated five times.

### Effect of inoculation time on the parasitism of *T*. *ostriniae*

Female *T*. *ostriniae* (five wasps/box) that had crossed within 24h were picked and inserted into a rectangular box (L×H×W = 15 cm×4.5 cm×10 cm) with an opening at the top, surrounded by and at the bottom of the box protected from light. The top opening was then sealed with a white-preferred color egg card and parasitized at a temperature of 25°C, humidity of 75%, and photoperiod of 14 L:10D for 8h, 16h, 24h, 32h, 40h, and 48h, respectively. After all parasite eggs were blackened and examined microscopically, the number of black eggs on the white-colored egg cards was counted. Each experiment was repeated five times.

### The impact of generational succession of parasitoid wasps on the parasitism of *T*. *ostriniae*

Female *T*. *ostriniae* (five wasps/box) that had crossed within 24h were picked and inserted into a rectangular box (L×H×W = 15 cm×4.5 cm×10 cm) with an opening at the top, surrounded by and at the bottom of the box protected from light. Then, the top opening was sealed with white—preferred color egg card, and parasitized at a temperature of 25°C, humidity of 75%, and a photoperiod of 14L:10D for 24h. Subsequently, the female wasps were removed, and the egg cards with the preferred color area were selected for further incubation. After all first-generation progeny emerged, we selected mated female *T*. *ostriniae* (5 wasps/box) within 24 h and repeated the above operations until the fifth generation of progeny. The number of black eggs on the white-colored egg cards after each generation of female wasps was recorded under a microscope. Each experiment was repeated five times.

### Influence of learning experience on the parasitism of *T*. *ostriniae*

Before the experiment, *T*. *ostriniae* underwent the following treatments: a. Inexperienced wasps emerged directly from white-background egg cards, having no exposure to the parent generation or during their development. b. Larval-learning wasps spent both the egg and larval stages within the eggs set against the preferred color background before ecolsion. c. Post-eclosion learning wasps, hatched from white-background egg cards, were introduced to preferred color egg cards and allowed to parasitize for 6h before removal, providing female wasps with oviposition experience on these preferred color cards. Female wasps (five wasps/box) were introduced into a rectangular box with the sides and bottom shielded from light, featuring an open top (L×H×W = 15 cm×4.5 cm×10 cm). The opening at the top was sealed with white egg cards, and parasitism was conducted for 24 hours under conditions of 25°C temperature, 75% humidity, and a 14L:10D light cycle. Following the removal of the female wasps, the parasitized eggs were incubated under the same conditions until all the eggs turned black. After completion, microscopic examination was conducted to count the number of black eggs on egg cards with various colored backgrounds.

### Statistical analysis

The data were statistically analyzed using SPSS software version 20.0. Before analysis, the data underwent the following transformation: normalized parasitism on white egg cards = parasitism on white egg cards/average parasitism on white egg cards, relative parasitism on colored egg cards = parasitism on colored egg cards / parasitism on white egg cards. The differences in parasitism between white and preferred color egg cards by *T*. *ostriniae* were analyzed using paired t-tests. The proportion of parasitized eggs in the white area = parasitism in the white area divided by total parasitism on white-preferred color egg cards, and the proportion of parasitized eggs in the preferred color area = parasitism in the preferred color area/total parasitism on white-preferred color egg cards. The proportions of parasitized eggs in white and colored areas were transformed using the arcsine square root transformation, according to the formula (y = arcsin(sqrt(n)) * 180 / π, where 1 ≥ n > 0)), to approximate a normal distribution. Shapiro–Wilk’s test was used to test for normality. Homogeneity of variances was tested using Levene’s test. One-way analysis of variance (ANOVA) was conducted with LSD (homogeneity of variance) and Dunnett T3 (nonheterogeneity of variance) post hoc tests to examine the differences in the parasitism of *T*. *ostriniae* on egg cards with different background colors, across generations on preferred color egg cards, and among individuals with varying learning experiences on preferred color egg cards. The parasitism rate on white egg cards served as a control. Linear regression was used to test the changes in the distribution proportion of *T*. *ostriniae* on the preferred color egg cards at different acceptance times. The significance level was set at *P* < 0.05.

## Results

### The yellow egg card affected the parasitism of female *T*. *ostriniae*

There was a significant difference in the relative amount of parasitism by female *T*. *ostriniae* on egg cards of different colors (F = 12.28, *P*<0.05). Female *T*. *ostriniae*, exhibited the highest parasitism on yellow egg cards and the lowest parasitism on red egg cards compared to white egg cards (*P*<0.05, Table 1). Additionally, the relative amount of parasitism by female *T*. *ostriniae* was not significantly different on white, green, blue, orange, and pink egg cards compared to white egg cards (*P*<0.05, Table 1).

**Table 1:** Preference of *T*. *ostriniae* to egg cards of different colors.

Egg cards of different colors	Relative amounts of parasitism		Minimum	Maximum
	Mean±SE	95% Confidence interval		
Red	0.23±0.04 c	0.12~0.34 0.63~1.08 0.76~1.05 0.75~1.04 0.56~1.24 1.09~1.39 0.92~1.08	0.14	0.31
Pink	0.85±0.09 b		0.69	1.24
Orange	0.91±0.06 b		0.79	1.16
Blue	0.89±0.06 b		0.67	1.02
Green	0.90±0.11 b		0.72	1.20
Yellow	1.24±0.05 a		1.12	1.33
White	1.00±0.04 b		0.76	1.56

### The inoculation time affected the parasitism of female *T*. *ostriniae*

**Fig 1:**
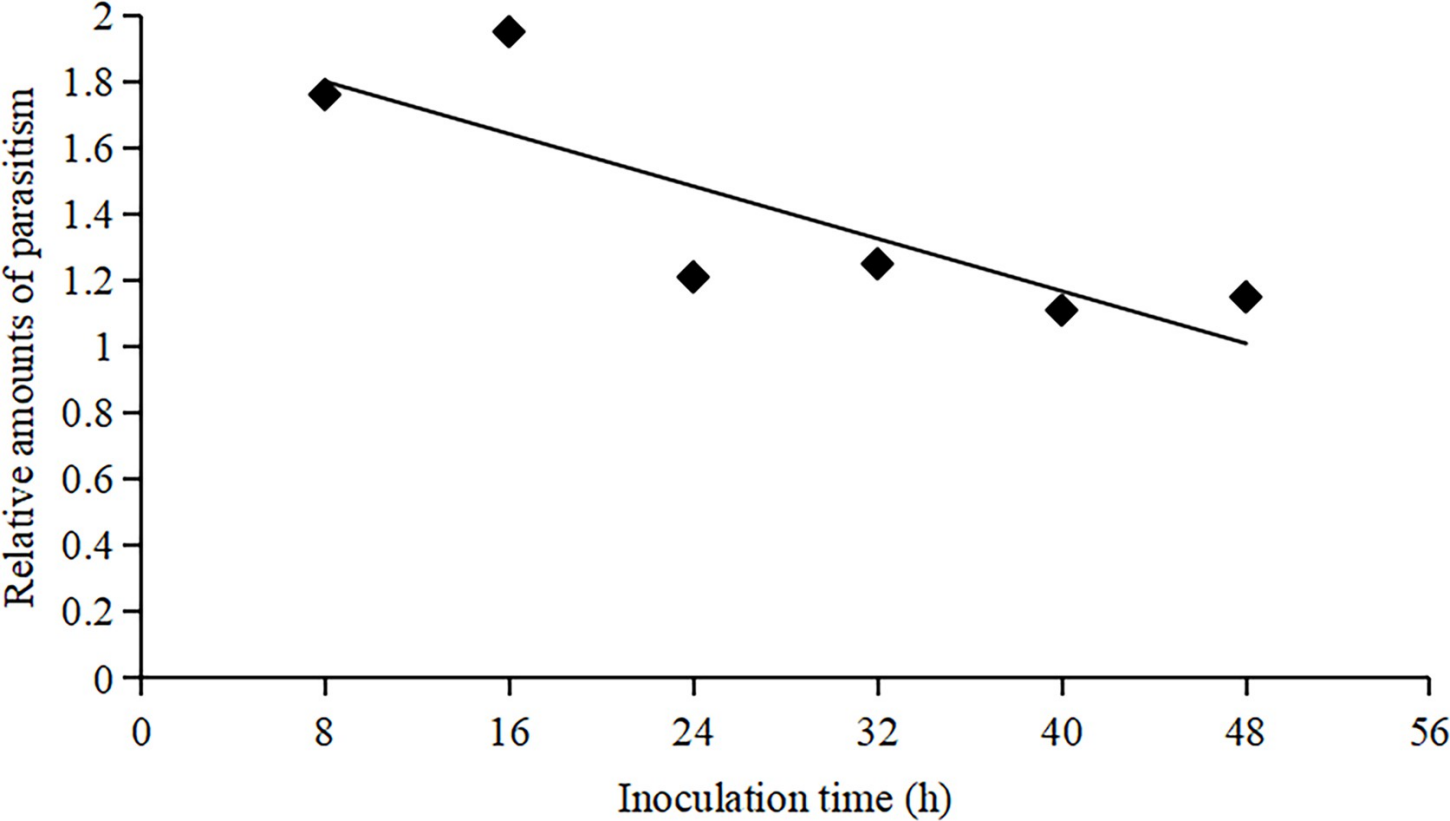
The change of relative amounts of parasitism to egg cards of yellow background in *T*. *ostriniae* at different inoculation times.

**Fig 2:**
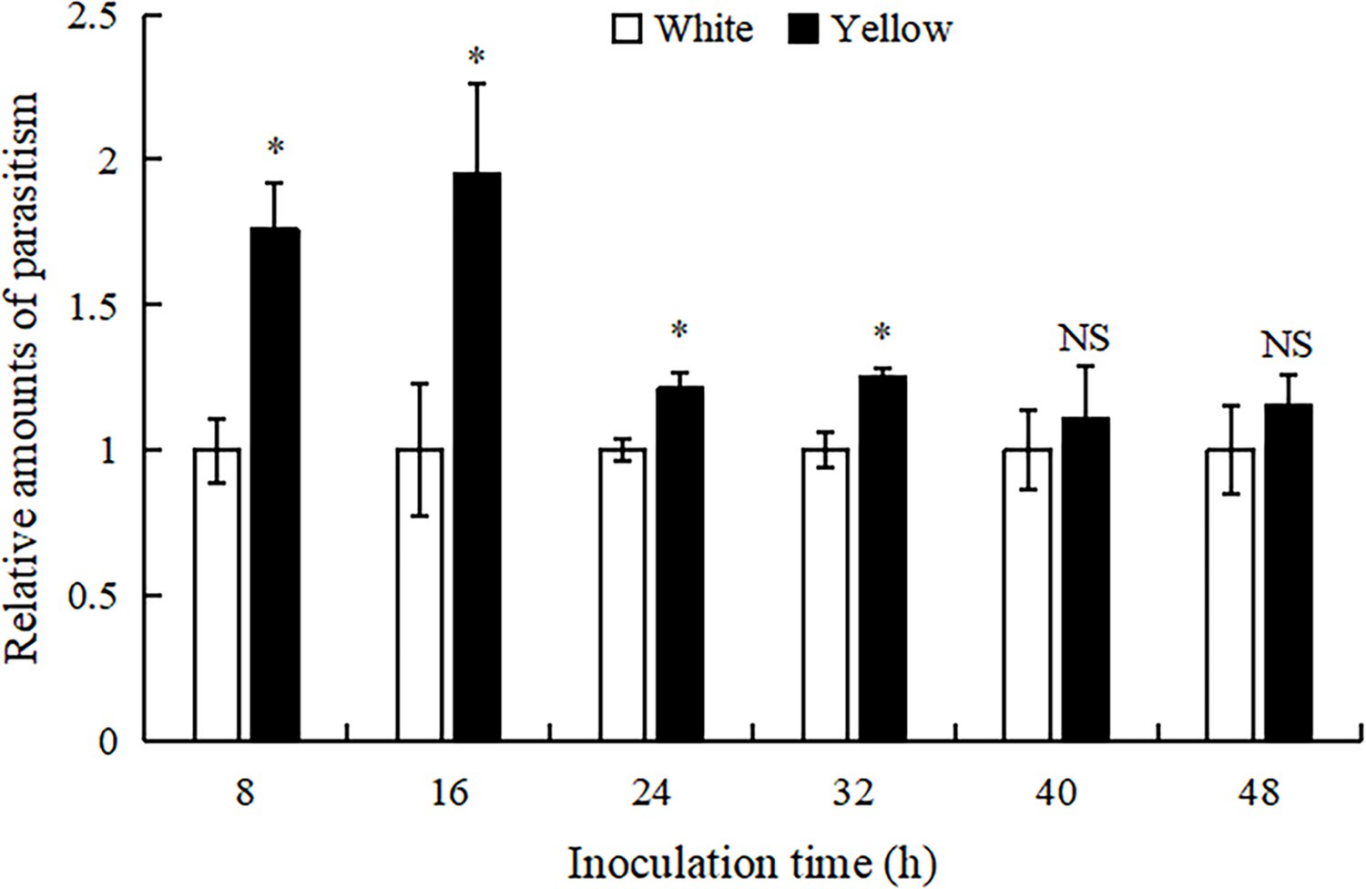
The differences in relative amounts of parasitism to yellow egg cards in *T*. *ostriniae* at the same inoculation time.

Significant differences were observed in the relative amounts of parasitism by female *T*. *ostriniae* on yellow egg cards at different inoculation times, with the highest parasitism occurring at 16h post-inoculation and the lowest parasitism at 40h post-inoculation (F = 4.07, *P*<0.05) (Table 2). Additionally, there was a generally negative linear correlation between the relative amount of parasitism on yellow egg cards and post-inoculation (F = 8.73, *P*<0.05) within 48h post-inoculation (Fig 1). Furthermore, the relative amount of parasitism by female *T*. *ostriniae* on yellow egg cards significantly increased at inoculation time of 8h, 16h, 24h, and 32h compared to white egg cards (*P*<0.05). However, there were no significant differences in the relative amounts of parasitism between yellow and white egg cards at inoculation times of 40h and 48h (*P*>0.05, Fig 2).

**Table 2:** Difference of parasitic quantity to egg cards of yellow background in *T*. *ostriniae* at different inoculation time.

Inoculation time (h)	Relative amounts of parasitism		Minimum	Maximum
	Mean±SE	95% Confidence interval		
8	1.76±0.16 ab	1.25~2.28 1.10~2.80 1.03~1.38 1.13~1.37 0.54~1.67 0.85~1.45	1.48	2.22
16	1.95±0.31 a		1.10	2.50
24	1.21±0.06 bc		1.06	1.43
32	1.25±0.03 bc		1.20	1.30
40	1.11±0.18 c		0.76	1.54
48	1.15±0.11 c		0.91	1.55

### Generations do not affect the parasitism of female *T*. *ostriniae*

**Fig 3:**
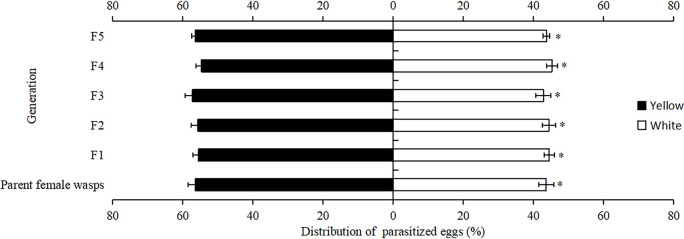
Distribution of parasitized eggs to yellow-white egg cards in *T*. *ostriniae* with different generations.

Subsequently, the effect of the number of generations on parasitism by female *T*. *ostriniae* was investigated. The analysis revealed no significant difference in the relative amount of parasitism between the parental generation and the subsequent five generations on yellow egg cards (F = 0.25, *P*>0.05; Table 3). However, within the same generation, the relative amount of parasitism by female *T*. *ostriniae* on yellow egg cards was significantly higher than that on white egg cards (*P*<0.05; Fig 3).

**Table 3:** Difference of parasitic quantity to yellow egg cards in *T*. *ostriniae* with different generations.

Generations	Relative amounts of parasitism		Minimum	Maximum
	Mean±SE	95% Confidence interval		
Parent female wasps	1.31±0.12	0.99~1.64	1.02	1.67
F1	1.26±0.08	1.05~1.47	1.03	1.47
F2	1.27±0.11	0.98~1.56	1.03	1.66
F3	1.35±0.11	1.00~1.70	1.04	1.52
F4	1.22±0.07	1.01~1.43	1.05	1.46
F5	1.30±0.05	1.15~1.44	1.14	1.44

### Learning experience does not affect the parasitism of female *T*. *ostriniae*

**Fig 4:**
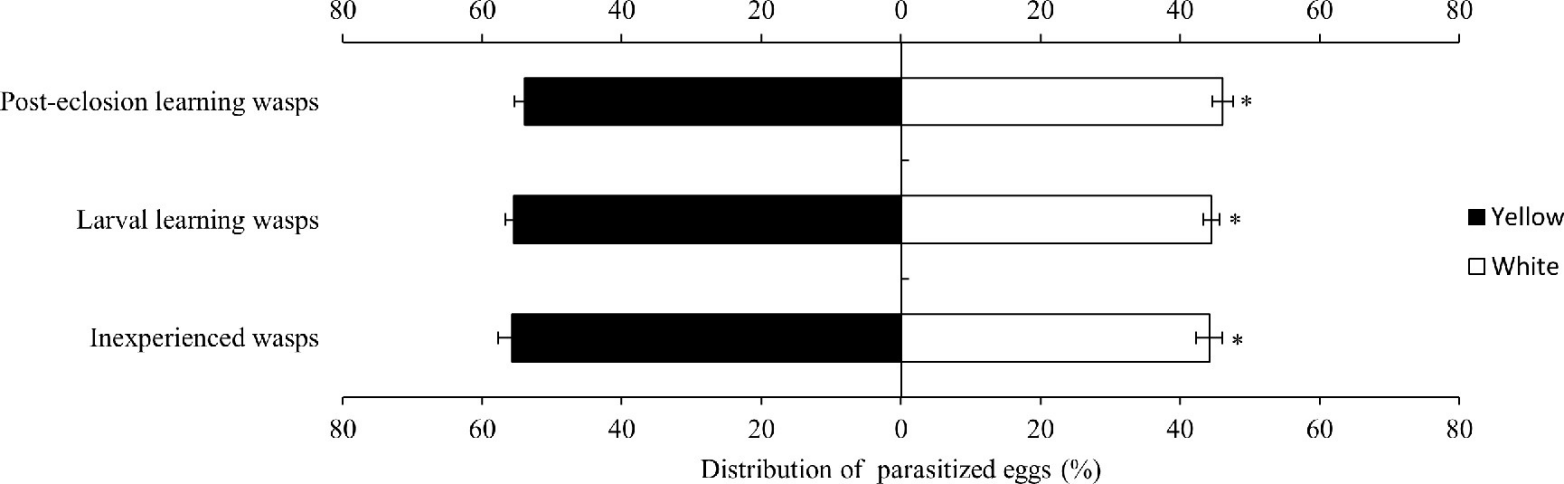
Distribution of parasitized eggs to yellow-white egg cards in *T*. *ostriniae* with different learning experiences.

Next, differences in the relative parasitism of female *T*. *ostriniae* on yellow egg cards were compared among the different learning experiences. The analysis showed no significant difference in the relative amount of parasitism on yellow egg cards by inexperienced, larval-learning, and post-eclosion-learning wasps (F = 0.25, *P*>0.05, Table 4). However, within the same learning experience, female *T*. *ostriniae* exhibited significantly higher relative amounts of parasitism on yellow egg cards to white egg cards (*P*<0.05; Fig 4).

**Table 4:** Difference of parasitism to yellow egg cards in *T*. *ostriniae* with different learning experience.

Learning experience	Relative amounts of parasitism		Minimum	Maximum
	Mean±SE	95% Confidence interval		
Inexperienced wasps	1.28±0.10	1.00~1.56	1.03	1.57
Larval learning wasps	1.26±0.08	1.05~1.47	1.03	1.47
Post-eclosion learning wasps	1.18±0.06	1.01~1.34	1.07	1.40

## Discussion

Color recognition plays a crucial role in the behavioral ecology of insects [10,12]. For parasitic insects like the *Trichogramma chilonis*, commonly known as the *trichogramma* wasp, the process of foraging for food, seeking mates, and locating hosts entail the reception and processing of a diverse array of color information from their surrounding environment [13,14]. A comprehensive understanding of the color preferences of *Trichogramma* wasps not only helps reveal the mechanisms of coevolution among plants, hosts, and predators but also has significant implications for guiding the scientific and rational release of these wasps in agricultural fields. This study aimed to investigate the color preferences of *T*. *ostriniae* across a spectrum of colors (white, yellow, orange, pink, red, blue, and green) and analyze the impact of inoculation time, generations, and learning experience on the parasitism on preferred egg cards. The findings revealed that female *T*. *ostriniae* exhibits a marked preference for yellow over other colors. Notably, the parasitism rate on yellow cards peaked at 16h post-inoculation. However, generation and learning experience did not affect the amount of parasitism of female *T*. *ostriniae* on yellow egg cards.

Insects possess a variety of receptor types in their eyes, enabling them to detect colors through inhibitory interactions between receptors sensitive to short and long wavelengths [15]. Many insect species have three types of photoreceptors: one sensitive to ultraviolet light (with a peak sensitivity near 350 nm), another to blue light (peaking around 440 nm), and a third to green light (with a maximum sensitivity at approximately 540 nm) [15]. For instance, the egg parasitoid wasp *T*. *ostriniae*, favors yellow over white, green, and black [10]. This predilection is believed to be an adaptive response to the yellow egg coloration of its primary lepidopteran host, *Ostrinia nubilalis*. Singh *et al*. examined the influence of differently colored cards used to affix host *C*. *cephalonica* eggs on the biological attributes of *T*. *chilonis* [16]. Their findings indicated that *T*. *chilonis* exhibited the highest rate of parasitism on yellow cards. Similarly, Manhar *et al*. discovered that *Trichogramma japonicum* also showed a pronounced preference for parasitism on yellow cards, followed by pink [17]. Additionally, two other egg parasitoid wasps, *Telenomus podisi* and *Trissolcus basalis*, have demonstrated a preference for yellow over green, brown, black, and white [18]. This predilection is also linked to the yellow egg color of their preferred hosts, *Euschistus heros* and *Nezara viridula*. Consistent with previous studies, our study found that *T*. *ostriniae* exhibited the highest parasitism rates on yellow cards compared to other colors. This preference may be attributed to the evolutionary adaptation of the corn borer *Trichogramma* to the egg color of its host [10]. In nature, Lepidoptera eggs exhibit a range of surface colors, making egg coloration a critical cue for *Trichogramma* wasps in host-searching and suitability assessments. The optimal host for the corn borer *Trichogramma* wasp is the eggs of the Asian corn borer, which typically display a white or yellowish-white coloration. Consequently, under the experimental conditions, females maintained a preference for yellow and white. The pronounced preference of *T*. *ostriniae* for yellow egg cards can be utilized to optimize field release strategies, thereby enhancing parasitism efficiency.

The rearing of parasitoid wasps, such as *T*. *ostriniae*, often involves high densities of female wasps and host eggs, leading to overcrowding that can have detrimental effects [19,20]. Inappropriate control of the inoculation time can result in superparasitism, which has several negative effects on the offspring, including reduced survival rates [21], prolonged developmental periods [22], diminished parasitic abilities [23], and smaller size [24], potentially affecting their overall capabilities [25,26]. Therefore, proper management of inoculation time is crucial for maintaining the quality of parasitoid wasps. Li *et al*. (2008) observed that inoculation time significantly influenced the reproduction of *T*. *ostriniae*, with the optimal production window being between 36 and 48 h. The most favorable outcomes occurred at 48 h, as evidenced by the highest eclosion rates and the maximum number of effective parasitisms per egg [27]. Our study revealed significant differences in the relative amounts of parasitism by *T*. *ostriniae* on yellow egg cards at various inoculation times, with peak parasitism occurring at 16 h post-inoculation and reaching a nadir at 40 h post-inoculation. Additionally, a general negative linear correlation was observed between the relative amounts of parasitism on yellow egg cards and the time elapsed post-inoculation within the initial 48-hour period. Furthermore, the relative parasitism rates of *T*. *ostriniae* on yellow egg cards were significantly higher at inoculation times of 8, 16, 24, and 32 h than those on white egg cards. Additionally, our investigation into the effect of the number of generations on *T*. *ostriniae* parasitism demonstrated a consistent pattern of parasitism across multiple generations. The absence of significant variation in parasitism rates between the parental generation and five subsequent generations when utilizing yellow egg cards suggests that the innate preference for yellow, potentially an adaptive trait for locating host eggs, is maintained in *T*. *ostriniae* and is not subject to rapid generational changes. These findings indicate that the highest parasitism rate occurs 16 hours post-inoculation, providing an optimized timing recommendation for field operations. Although the effects of generation and learning experience on parasitism rates were not significant, yellow egg cards consistently exhibited high parasitism rates under various environmental conditions, further confirming their reliability in practical applications. By effectively utilizing *T*.*ostriniae*, farmers can reduce their reliance on chemical pesticides, lower production costs, and minimize environmental pollution caused by chemical substances.

Furthermore, the strength of this color preference was supported by the lack of significant disparities in parasitism rates among naive, larval-trained, and post-emergence-trained wasps. This finding indicates that preferences are not substantially altered by experiential learning. This result contrasts with previous research, suggesting that parasitic wasps with learning experience could improve host-search efficiency and parasitism rates through associative learning. For instance, female *Trichogramma evanescens* with oviposition experience demonstrated a notable reduction in the time taken to locate hosts compared to their inexperienced counterparts [28]. Similarly, *Sclerodermus pujpariae* Yang et Yao exhibited a significant decrease in the time required to locate hosts after initial exposure [29]. Wang *et al*. (2011) observed that *Scleroderma guani* Xiao et Wu exhibited a pronounced preference for rust-colored longhorn beetles after prior exposure [30]. Additional studies are needed to validate these findings.

This study had several limitations. First, the preference of *T*. *ostriniae* for specific colors was assessed only in indoor environments. However, in natural settings, these parasitoids may encounter a more diverse and variable range of colors due to biotic and abiotic environmental factors. Therefore, future field experiments should be conducted to objectively and accurately compare and evaluate the color preferences of *T*. *ostriniae*. Secondly, this study did not investigate whether the parasitism rates of *T*. *ostriniae* on egg cards of other colors were influenced by factors such as inoculation time, generation of introduction, and learning experience. Third, the study did not consider other environmental factors that might affect the behavior of these parasitoid wasps, such as food availability and flight behavior, nor did it examine which sensory cues T. ostriniae uses to detect and differentiate colors or whether there are physiological or behavioral responses to different colors. Future, larger-scale studies are needed to explore these aspects in greater depth.

In summary, female *T*. *ostriniae* demonstrated the strongest parasitic preference for yellow egg cards and the weakest preference for red egg cards. Additionally, the relative parasitism of female *T*. *ostriniae* on yellow egg cards peaked at 16 h post-exposure, with a general negative linear correlation between relative parasitism on yellow egg cards and exposure time observed within the initial 48 h post-exposure. However, we observed no significant difference in the relative parasitism of yellow egg cards between parents and offspring, and short-term learning did not influence the preference of female *T*. *ostriniae* for yellow cards. These findings suggest that optimizing release strategies, such as selecting yellow egg cards and determining appropriate inoculation times, can significantly enhance parasitic efficiency. This approach not only contributes to the sustainability of agricultural production but also offers multiple benefits to farmers and the environment. Future research should explore the application effects under various environmental and crop conditions to maximize the biocontrol potential of *T*.*ostriniae*.

## Supporting information

S1 Raw data (XLSX)

S1 Table (DOCX)
