# Supplementary material for: Influence of egg card color preference, inoculation time, generational succession, and learning experience on the parasitism of Trichogramma ostriniae
Source: PLoS One. 2025 Feb 21;20(2):e0315970. doi: 10.1371/journal.pone.0315970 (PMC11844909; doi:10.1371/journal.pone.0315970)
Supplement: S1 Table — (DOCX) [file pone.0315970.s002.docx]

**Table S1 The color parameters of different color card**

| Color | Luminosity | Range from red to green | Range from yellow to blue |
| --- | --- | --- | --- |
| White | 93.48 ± 0.03 | 2.607 ± 0.02 | -10.08 ± 0.10 |
| Yellow | 93.33 ± 0.02 | -4.20 ± 0.02 | 34.51 ± 0.12 |
| Orange | 89.85 ± 0.02 | 13.34 ± 0.06 | 23.62 ± 0.06 |
| Pink | 79.19 ± 0.06 | 30.82 ± 0.09 | -0.54 ± 0.03 |
| Red | 57.33 ± 0.05 | 61.34 ± 0.03 | 39.04 ± 0.04 |
| Blue | 83.96 ± 0.03 | -14.86 ± 0.02 | -12.32 ± 0.07 |
| Green | 87.91 ± 0.03 | 2.61 ± 0.02 | -10.08 ± 0.10 |
